# Proteasomal Degradation of Mcl-1 by Maritoclax Induces Apoptosis and Enhances the Efficacy of ABT-737 in Melanoma Cells

**DOI:** 10.1371/journal.pone.0078570

**Published:** 2013-11-04

**Authors:** Manoj K. Pandey, Krishne Gowda, Kenichiro Doi, Arun K. Sharma, Hong-Gang Wang, Shantu Amin

**Affiliations:** Department of Pharmacology, Penn State College of Medicine, Hershey, Pennsylvania, United States of America; University of Colorado, School of Medicine, United States of America

## Abstract

**Background and purpose:**

Metastatic melanoma remains one of the most invasive and highly drug resistant cancers. The over expression of anti-apoptotic protein Mcl-1 has been associated with inferior survival, poor prognosis and chemoresistance of malignant melanoma. A BH3 mimetic, ABT-737, has demonstrated efficacy in several forms of cancers. However, the efficacy of ABT-737 depends on Mcl-1. Because the over expression of Mcl-1 is frequently observed in melanoma, specifically targeting of Mcl-1 may overcome the resistance of ABT-737. In this study, we investigated the effects of Maritoclax, a novel Mcl-1-selective inhibitor, alone and in combination with ABT-737, on the survival of human melanoma cells.

**Experimental approach:**

For cell viability assessment we performed MTT assay. Apoptosis was determined using western blot and flow cytometric analysis.

**Key results:**

The treatment of Maritoclax reduced the cell viability of melanoma cells with an IC_50_ of between 2.2–5.0 µM. Further, treatment of melanoma cells with Maritoclax showed significant decrease in Mcl-1 expression. We found that Maritoclax was able to induce apoptosis in melanoma cells in a caspase-dependent manner. Moreover, Maritoclax induced Mcl-1 degradation via the proteasome system, which was associated with its pro-apoptotic activity. We also found that Maritoclax treatment increased mitochondrial translocation of Bim and Bmf. Importantly, Maritoclax markedly enhanced the efficacy of ABT-737 against melanoma cells in both two- and three-dimensional spheroids.

**Conclusions and implications:**

Taken together, these results suggest that targeting of Mcl-1 by Maritoclax may represent a new therapeutic strategy for melanoma treatment that warrants further investigation as a single therapy or in combination with other agents such as Bcl-2 inhibitors.

## Introduction

Melanoma is the most aggressive type of skin cancer, with high mortality. Despite a wide variety of available therapeutic strategies [1] the average survival rate is still poor and generally varies from 6-12 months [2]. Targeted therapies directed against PI3K/AKT [3], BRAF-V600E[4], and mutant KIT[5], have produced major preclinical or clinical responses. However, these responses are not typically complete or durable. For example clinical testing of imatinib has been limited to a subset of patients harboring certain mutations in KIT [5], the majority of patients administered with PLX4032 (vemurafenib), a structural analogue of PLX4720, specific drug against mutant B-RAF exhibit a partial response [4], and the alkylating agent dacarbazine (DTIC), the FDA-approved drug for the treatment of malignant melanoma as a single agent allows complete remissions only on 5–10% of patients. Thus, there is an urgent need of new therapeutic invention for metastatic melanoma.

The identification of molecules involved in the regulation and execution of apoptosis, and their alteration in melanoma, have provided new insights into the molecular basis for melanoma chemoresistance [6]. Thus, activation of apoptotic pathways may be an alternative antitumor strategy and would be valuable to overcome *de novo* or acquired resistance to conventional chemotherapy. Along these lines, Bcl-2 family, in particular, has attracted much attention [7]. This family can be divided into three groups: anti-apoptotic proteins, including proteins such as Bcl-2, Bcl-xL, Bcl-w, and Mcl-1; multi-domain pro-apoptotic proteins Bax and Bak; and pro-apoptotic BH3-only proteins, including Noxa, Bim, Bid, Bad, Bmf, and Bik. Interactions between members of these three factions of the Bcl-2 family dictate whether a cell lives or dies. When BH3-only proteins have been activated, for example, in response to DNA damage, they can bind via their BH3 domain to a groove on their pro-survival relatives. How the BH3-only and Bcl-2-like proteins control the activation of Bax and Bak, however, remains to be better understood. Recent studies have suggested that Bak is held in check solely by Mcl-1 and Bcl-xL and induces apoptosis only if freed from both [8]. Most attention has focused on Bax. The BH3-only proteins therefore play the key role of determining whether Mcl-1 and Bcl-xL are available to sequester Bak. Studies by Willis et al, 2005, have emphasized that Noxa not only displaces Bak from Mcl-1 but also promotes the proteasome-dependent degradation of Mcl-1 [8]. Thus, Noxa acts to inactivate Mcl-1 by binding and triggering its destruction. Among anti-apoptotic family, the overexpression of Mcl-1 has been shown to be associated with anoikis-, autophagy-resistance, and poor prognosis of various tumors including melanoma [9]. Furthermore, observations of increased Mcl-1 and Bcl-xL levels in thin primary melanomas as well as in metastatic malignant melanomas but not in benign nevi, suggest that up regulation of these proteins represents an early event associated with malignant transformation [10]–[12]. The suppression of Mcl-1 inhibited the proliferation of a wide variety of human tumor cells, including prostate cancer [13], pancreatic cancer [14], small-cell lung cancer [15], ovarian cancer [16], chronic lymphocytic leukemia [17], hepatoma [18], leukemia [19], chronic lymphocytic leukemia [20], breast carcinoma [21], and melanoma [22], [23]. Thus, Mcl-1 may play a critical role in the initiation of melanoma development and seems to be a suitable molecular target to enhance chemo-sensitivity of this dreaded disease [24]–[26]. The prior studies suggest that reduction of Mcl-1 or induction of NOXA sensitized cells to chemotherapeutic drugs including ABT-737 in a variety of tumor models [23], [24], [26], [27].

Over the last few years, several small molecule Bcl-2 inhibitors have been synthesized as BH3 mimetic and some of these molecules have entered clinical trials [28]. To date, the most potent and selective small-molecule Bcl-2 inhibitors are ABT-737, ABT-263 (the orally active analog of ABT-737), and ABT-199. ABT-737 is a BH3-mimetic with a similar binding profile to the pro-apoptotic BH3-only protein BAD; it strongly inhibits the activity of Bcl-2, Bcl-xL, and BCL-w but not Mcl-1 or Bfl-1/A1, for which it has low affinity [29], [30]. ABT-737 is effective as a single agent in preclinical models of lymphomas and small-cell lung carcinomas (SCLC) with low Mcl-1 expression [29], [31], [32]. In recent years, it has been learnt that the effectiveness of BAD BH3-mimetics are dependent on the Mcl-1 expression and studies suggest that cancer cells can quickly acquire resistance to ABT-737 by up-regulation of Mcl-1 [24], [33]. Furthermore, either suppression of Mcl-1 or induction of NOXA sensitizes cells to ABT-737 in a variety of tumor models, suggesting that a treatment regime combining ABT-737 with a Mcl-1-specific inhibitor may be necessary to overcome the resistance against ABT-737 [24], [29], [30], [34], [35], as it is necessary to neutralize both arms of the anti-apoptotic Bcl-2 family (Bcl-2/Bcl-xL and Mcl-1) for apoptosis to occur in many cancer cell types.

Because Mcl-1 is associated with melanoma progression, poor prognosis, and chemoresistance, we postulated that suppression of Mcl-1 would potentiate the apoptotic effects of ABT-737 in metastatic melanoma. Recently, Marinopyrrole A (Maritoclax) has been identified as a Mcl-1 selective inhibitor [19]. In this report, we studied the effects of Maritoclax on Mcl-1 expression and associated apoptotic response in melanoma cells in 2- and 3-dimensional models. We found that Maritoclax specifically suppresses the expression of Mcl-1. Moreover, Maritoclax induces Mcl-1 degradation via the proteasome system, which is associated with the pro-apoptotic activity of Maritoclax. We found that Maritoclax treatment increased Bim and Bmf translocation to mitochondria. We investigated the extent to which Mcl-1 affects the sensitivity of melanoma cells to ABT-737 and thus whether combination therapy targeting Mcl-1 and Bcl-2 has a promise in the context of melanoma.

## Methods

### Antibodies and Compounds

Antibodies were obtained from the following sources: rabbit anti-Mcl-1 (Cell Signaling, 4572); rabbit anti-Bcl-xL (Cell Signaling 2762), rabbit polyclonal anti-Bak (Cell Signaling 3814), rabbit monoclonal anti-Bax (Cell Signaling 5023), rabbit polyclonal anti-Bmf (Cell Signaling 5889), rabbit polyclonal anti-Bim (Cell Signaling 2819), rabbit polyclonal anti-caspase-3 (Cell signaling 9662), rabbit polyclonal anti-PARP (Cell Signaling 9542); rabbit anti-GAPDH (Sigma-Aldrich G9545), mouse anti-Bcl-2 (Santa Cruz SC-7382), rabbit anti-Bax (Santa Cruz SC-493), mouse monoclonal anti-NOXA (Thermo Scientific MA1-41000). ABT-737 was obtained from Selleck Chemicals (Houston, TX, USA). Thiazolyl Blue Tetrazolium Bromide (MTT) was purchased from Sigma-Aldrich. LIVE/DEAD viability/cytotoxicity kit, for mammalian cells was purchased from Life technologies (L-3224). Maritoclax was synthesized as racemic mixture as previously described [19].

### Cell Culture

A375M, 1205Lu cell lines maintained in DMEM medium, UACC903 cells were maintained in RPMI 1640 medium supplemented with 10% fetal bovine serum (FBS) and 100 units/ml penicillin, 100 µg/ml streptomycin at 37°C and 5% CO_2_. These cells were obtained from ATCC. The cells were routinely screened for mycoplasma using Hoechst 33258 staining.

### Cell Viability Assay

The effects of Maritoclax alone or in combination with ABT-737 on the cell viability were determined by the MTT uptake method as previously described [36]. Briefly, 2500 cells were incubated with various concentrations of Maritoclax or ABT-737 or together in triplicate in a 96-well plate for 24 h and 48h at 37°C. An MTT solution was added to each well and incubated for 3 h at 37°C. After 3h formazon crystals were dissolved by DMSO and optical density was measured at 570 nm using a 96-well multiscanner (Dynex Technologies, MRX Revelation; Chantilly, VA, USA). Backgrounds were subtracted at 630 nm. IC_50_ values were calculated by non-linear regression analysis using Prism software.

### Live/Dead assay

To measure cell death, we used the Live/Dead assay (Life technologies, USA), which determines intracellular esterase activity and plasma membrane integrity [36]. Non-fluorescent polyanionic dye calcein AM is retained by live cells and by enzymatic conversion (esterase) it becomes fluorescent, produces intense green fluorescence in live cells. Ethidium homodimer enters cells with damaged membranes and binds to nucleic acids, thereby producing a bright red fluorescence in dead cells. Briefly, 2×10^5^ cells were incubated with either Maritoclax or ABT-737 or combination for 24 h at 37°C. Cells were stained with the Live-Dead reagent (5 µM ethidium homodimer and 5 µM calcein-AM) and analyzed by flow cytometry according to the manufacture’s protocols.

### Annexin-V Assay

The annexin-V assay uses the binding properties of annexin-V to detect the rapid translocation and accumulation of the membrane phospholipid phosphatidyl-serine from the cytoplasmic membrane interface to the extracellular surface, an indicator of early apoptosis. We detected this loss of membrane asymmetry using an annexin-V antibody conjugated with the fluorescein isothiocyanate fluorescence dye. Briefly, 5×10^5^ cells were treated with either Maritoclax or ABT-737 or combination for 24h at 37°C, and subjected to annexin-V staining. The cells were washed in phosphate-buffered saline, resuspended in 100 µl of binding buffer containing a fluorescein isothiocyanate-conjugated anti-annexin V antibody, and analyzed with a flow cytometer (FACSCalibur, BD Biosciences).

### Subcellular fractionation

Subcellular fractionation was performed by using mitochondria isolation kit (Thermo Scientific, USA, 89874). Briefly, 20×10^6^ cells were treated with either Maritoclax or MG132 or combination for 12h at 37°C. Same numbers of cells were treated with actin polymerization inhibitor cytochalasin D for 3h at 37°C. After, incubation cells were harvested and washed in ice-cold PBS and resuspended in buffer A, on ice for 2 min. Subsequent steps were followed according to the manufacture’s protocol and mitochondrial pellets were lysed in 2% Chaps buffer for immunoblot analysis.

### Western blot analysis

To determine the effect of Maritoclax and ABT-737 on Mcl-1 and associated apoptotic pathways, whole-cell extracts were prepared by subjecting Maritoclax- or ABT-737-treated cells to lysis in 1% Chaps buffer supplemented with protease and phosphatase inhibitor cocktails as described before [19]. Lysates were spun at 15,000 rpm for 10 minutes to remove insoluble material. Supernatant were collected and kept at -80°C. Lysates were resolved by SDS-PAGE. After electrophoresis, the proteins were electro-transferred to PVDF membranes, blotted with the relevant antibody, and detected by enhanced chemiluminescence reagent. To save time and precious samples, sequential detections of different proteins were performed on same membrane by using Restore Western blot stripping buffer (Thermo Scientific, USA). All critical blots experiments were repeated at least two-three times.

### Melanoma 3D spheroid assays

Among 3D cultures, 3D alginate scaffold has advantages being an animal-free product with significant stability at room temperature [37]. 3D alginate scaffold is a non-toxic and biodegradable ready-to-use sponge made from lyophilized alginate gel, which supports a cell culture model resembling normal cell characteristics and morphology. The multicellular spheroids can be easily visualized under the microscope to monitor the growth characteristics [38].

We have first optimized and developed the *in vitro* alginate scaffold 3D tumor model using UACC 903 human melanoma cells. After optimization, 0.50×10^6^ UACC903 cells were incorporated in each well of the 6 well plates. Then 2 ml of cell suspension containing firming buffer was added to the 3D alginate scaffold cultures system. The growth of tumor spheroids was assessed by observing formation of the spheroids in 3D alginate scaffold well. Spheroids were grown up to 7 days and then treated with either ABT-737 (5.0 µM) or Maritoclax (2.0 µM) alone or in combination for three days. The spheroids growing in the 3D AlgiMatrix could be observed without dissolving the matrix or removing media. At 0d, 7d and 10d post cell seeding, images were taken using an inverted microscope; the size and numbers of spheroids were determined. From each well an average of 12-15 fields were used for these measurements. Spheroid sizes were quantified by image analysis using ImageJ software (NIH, Bethesda, MD).

### Isolation of spheroids from AlgiMatrix

At the end of the experiments spheroids were isolated from matrix using AlgiMatrix dissolving buffer (Catalogue no A11340-01, Life technologies) following manufactures’ protocol. Dissolving buffer a non-enzymatic solution, this dissolves the matrix within a few minutes but leaves the spheroids intact for further processing and/or analysis. In brief, all residual liquid media from the vessel containing the sponge was carefully aspirated using a pipette. 5 ml of dissolving buffer was added directly onto the sponge and incubated for 5-10 minutes, while examining the sponge under a microscope. After incubation the contents of the well were transferred to a sterile centrifuge tube and equal amount culture medium was added to the centrifuge tube(s). Tubes were centrifuged at 200 × g for 5 minutes to pellet the released cells. Pellets were washed with dissolving buffer to remove any debris. The supernatant was removed and pellets were carefully resuspended in PBS. The isolated spheroids were stained with 4 mmol/L calcein-AM and 2 mmol/L ethidium bromide (Invitrogen) for 1 hour at 37°C. Images were taken using a Nikon-300 inverted fluorescence microscope.

### Clonogenic survival assay

Clonogenic survival assay was performed as described earlier [39]. In brief, UACC 903 melanoma cells were seeded in the 6 well plates. Cells were treated with vehicle control (DMSO), Maritoclax, ABT-737 or in combination for 24h. After 24h, cells were trypsinized, and counted. Vehicle control cells were replated in 100-mm culture dishes at densities of 100, 300 cells per plate in triplicate. The cells treated with either Maritoclax or ABT-737 or in combination were reseeded in a 100 mm dish at densities of 1000, 3000 cells per plate in triplicate. Cells were incubated for 10 to 14 days and then fixed with 10% methanol/10% acetic acid and stained with 0.4% crystal violet. Colonies containing more than 50 cells were counted. The plating efficiencies were determined for each treatment and normalized to controls. The average normalized surviving fraction from triplicates and the S.E.M. were reported.

### Statistical analysis

Statistical significance of the results was analyzed by Student's *t*-tail test using GraphPad Prism 3.0 software.

## Results

### Maritoclax induces apoptosis of melanoma cells

**Figure 1:**
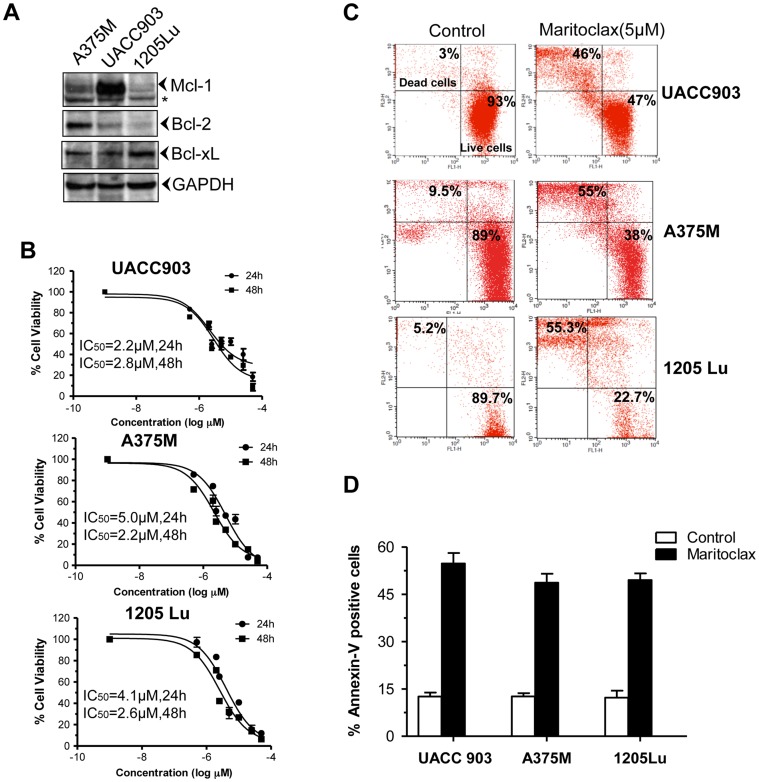
Expression of anti-apoptotic proteins and effects of Maritoclax on cell survival in melanoma cells. ***A,*** Melanoma cells were harvested, lysed, and fractionated. The Western blot analysis was performed using mentioned antibodies and same blots were re-probed with either other antibodies or with anti-GAPDH to access loading. ***B,*** Melanoma cells were treated with increasing concentrations of Maritoclax (0.1–30 µM) for 24 and 48h. Cell viability was then analyzed by the MTT assay. IC_50_ values were calculated by non-linear regression analysis. ***C-D***, Melanoma cells were treated with 5.0 µM of Maritoclax for 24h and apoptosis was determined by Live/Dead assay and Annexin-V- FITC binding through flow cytometry as described under Materials and Methods.

We first investigated the expression levels of anti-apoptotic Bcl-2 family protein in melanoma A375M, UACC903, and 1205Lu cells. Interestingly, higher expression of Mcl-1 was observed in UACC903 cells. Expression level of Bcl-2 was found higher in A375M cells, whereas relatively higher amount of Bcl-xL was observed in 1205Lu cells (Fig. 1A). We determined the effects of Maritoclax on cell viability of melanoma cells by two different methods. As depicted in Fig. 1B, treatment of Maritoclax reduced the cell viabilities of melanoma cells with an IC_50_ between 2.2–5.0 µM. We also determined the cytotoxic effects of Maritoclax by live-dead assays in melanoma cells as reflected by Fig. 1C, almost 40–75% cell death was observed in Maritoclax treated cells. In addition, annexin-V staining showed that Maritoclax up-regulated early apoptosis in melanoma cells (Fig. 1D). Together these results indicated that Maritoclax induces apoptosis in melanoma cells.

Since our results indicate that UACC903 cells are more sensitive to Maritoclax and show high expression of Mcl-1, we further investigated effects of Maritoclax on this cell line.

### Maritoclax specifically inhibits Mcl-1 expression of UACC903 melanoma cells

**Figure 2:**
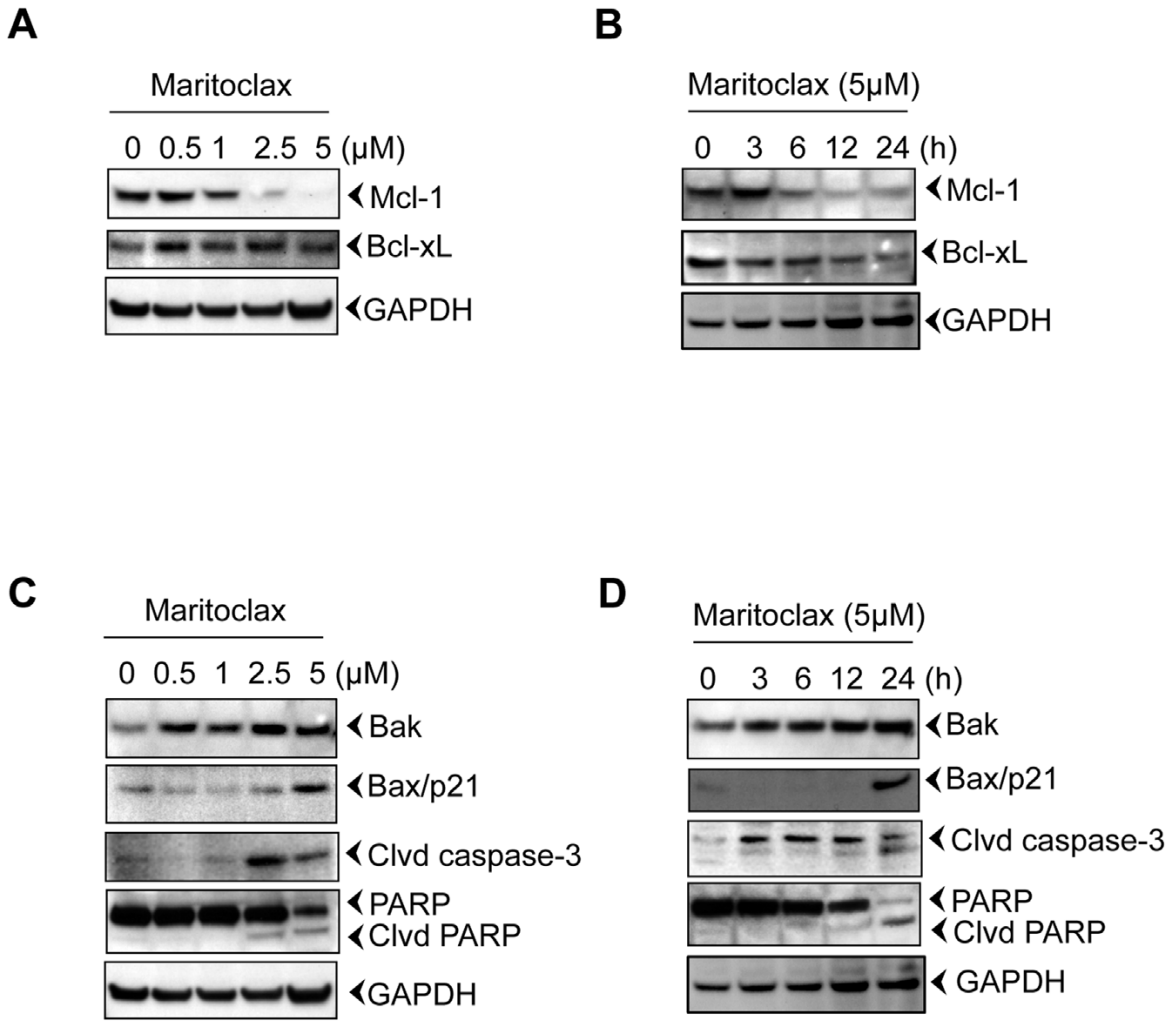
Down regulation of Mcl-1 induces apoptosis in melanoma cells. Melanoma cells were incubated with either indicated amount of Maritoclax for 24h to access dose response or with 5 µM Maritoclax for mentioned time points. After incubation, cells were harvested; total lysates were prepared, and fractionated. The Western blot analysis was performed using mentioned antibodies and same blots were re-probed with either other antibodies or with anti-GAPDH to access loading. The results shown are representative of two independent experiments. ***A-B,*** Maritoclax had no effect on Bcl-2 and Bcl-xL. ***C-D,*** Degradation of Mcl-1 is associated with apoptosis.

Since melanoma cells are dependent on Mcl-1 for survival [11], [40] and Maritoclax is a selective inhibitor of Mcl-1 [19], we investigated the effects of Maritoclax on Mcl-1 expression. As shown in Fig. 2A & 2B, Maritoclax suppressed Mcl-1 dose and time dependently. As expected, we found that response of Maritoclax on Mcl-1 was specific, since it did not affect Bcl-xL expression (Fig. 2A & B). Our results further support the report about the specificity of Maritoclax on Mcl-1 in leukemia cells [19]. These results suggest that Maritoclax specifically inhibits Mcl-1 expression of melanoma cells.

### Maritoclax induces caspase-mediated apoptosis by degradation of Mcl-1 protein

Because our results suggest that Maritoclax inhibits anti-apoptotic Mcl-1 expression; we were interested to investigate effects of Maritoclax on apoptosis. Our results show that inhibition of Mcl-1 does induce the Bak expression (Fig. 2C & D). Similarly, Maritoclax induced Bax expression, however as compared to Bak, maximum expression of Bax was observed in 24h with 5.0 µM treatment (Fig. 2C & D). Further, Maritoclax induced caspase-3 activation in UACC903 cells as demonstrated by procaspase-3 processing and PARP cleavage (Fig. 2C & D).

### Maritoclax induces proteasomal degradation of Mcl-1 and releases Bim and Bmf from the cytoskeleton

**Figure 3:**
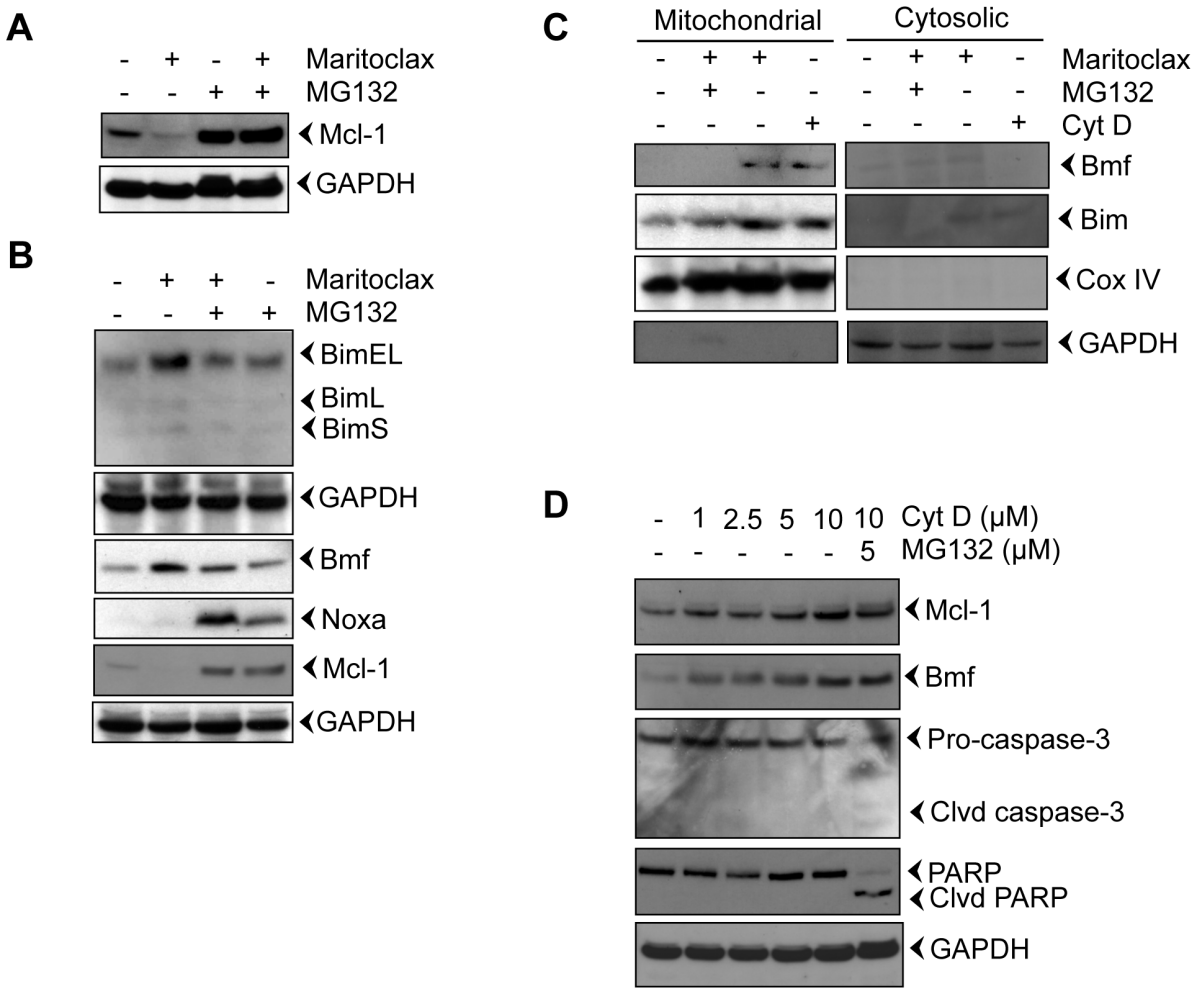
Maritoclax degrades Mcl-1 by proteasomal activation and releases Bim and Bmf from the cytoskeleton. ***A-B,*** Melanoma cells were treated with 5.0 µM of Maritoclax alone or a combination of 5.0 µM Maritoclax and 5 µM MG132 for 12 h and levels of Mcl-1, Bmf, Bim, and Noxa were assessed by immunoblot analysis. ***C,*** Melanoma cells were treated with Maritoclax (5.0 µM, 12h) alone, a combination of Maritoclax (5.0 µM) and MG132 (5.0 µM) for 12 h, and cytochalasin D (10 µM, 3h) alone. Subcellular fractions were prepared. Levels of Bmf, Bim, Cox IV, and GAPDH were assessed by immunoblot analysis. ***D,*** Melanoma cells were treated with either indicated amount of cytochalasin D or MG132 for 12h. Whole cell extracts were prepared. Levels of Bmf, Mcl-1, Caspase-3, and PARP cleavage were assessed by immunoblot analysis. Loading was confirmed by GAPDH.

We investigated whether addition of the proteasome inhibitor MG132 attenuates Maritoclax-mediated Mcl-1 degradation. Fig. 3A reveals that degradation of Mcl-1 is mediated through proteasomes, suggesting that the pro-apoptotic effect of Maritoclax is attributed to its ability to induce proteasomal degradation of Mcl-1 as reported earlier [19]. In healthy cells, binding to dynein light chains sequesters Bim and Bmf to the cytoskeleton. In response to cytotoxic stimuli, that induce cytoskeletal rearrangements, Bim and Bmf may act as stress sensors, released from the cytoskeleton, and induce the activation of Bak and Bax. We found that Maritoclax induced the levels of BimEL and Bmf (Fig. 3B); other isoforms of Bim were not induced. The activation of Bmf and BimEL was associated with translocation to mitochondria (Fig. 3C). Addition of MG132 suppressed the levels of Bmf and BimEL and prevented the release of Bmf and BimEL from the cytoskeleton to mitochondria (Fig. 3B and C). We further investigated whether the effects of Maritoclax on Bim and Bmf are primary or the downstream event of Mcl-1 degradation. We treated cells with increasing dose of cytochalasin D for 12h and effects on Bmf, Mcl-1, caspase activation, and PARP degradation was assessed (Fig. 3D). As expected we found dose dependent activation of Bmf (Fig. 3D). Interestingly, cytochalasin D neither facilitate degradation of Mcl-1, nor caspase activation and PARP degradation, suggesting that activation of Bmf, Bim followed by caspase activation are downstream events of Mcl-1 degradation.

Taken together, our results suggest that Maritoclax degrades Mcl-1 by proteasomal activation, and this degradation facilitates release of Bim and Bmf from cytoskeleton, activates caspase and degradation of PARP.

### Maritoclax potentiates apoptotic effects of ABT-737 in melanoma cells

**Figure 4:**
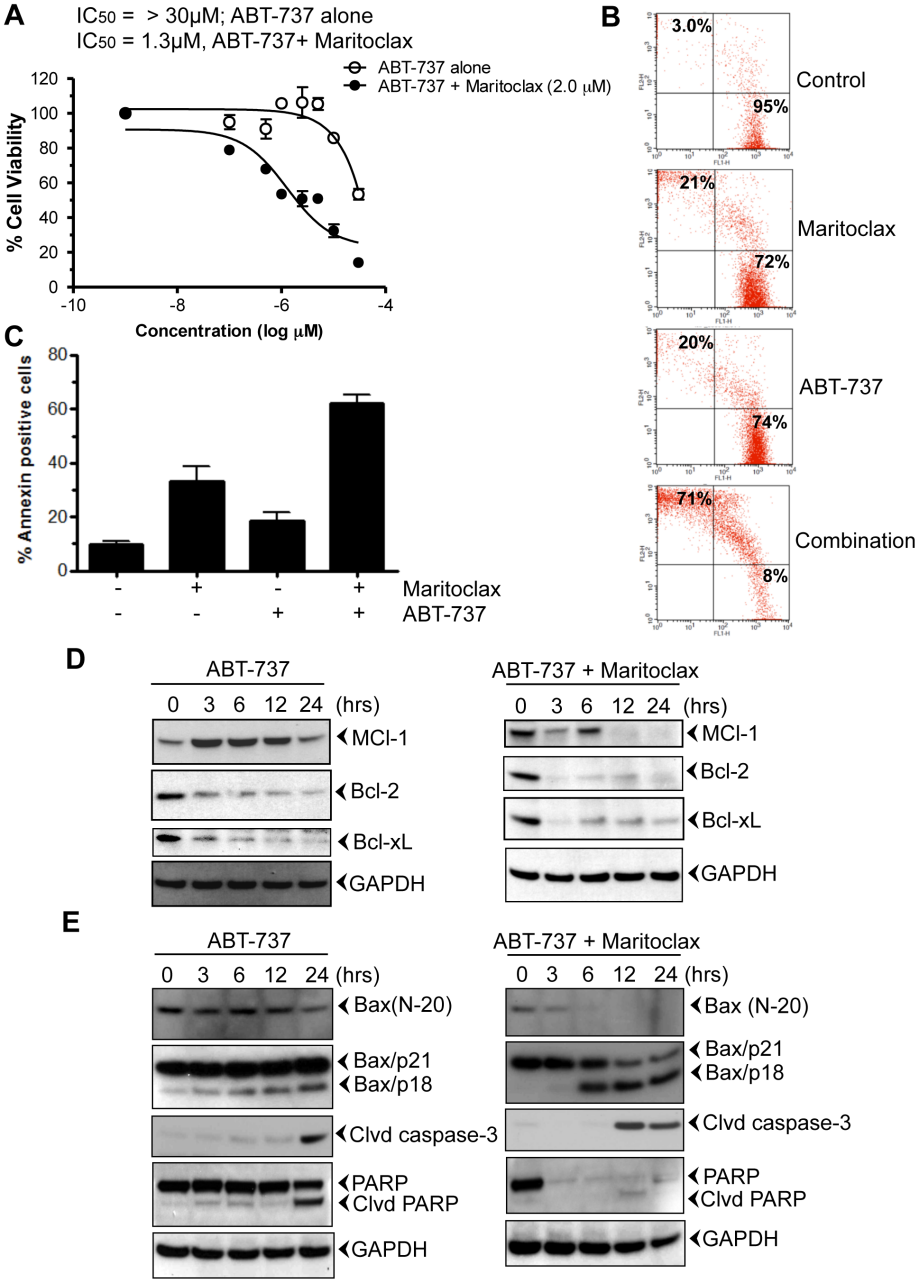
Maritoclax sensitizes melanoma cells to ABT-737. ***A***, UACC903 cells were treated with increasing amount of ABT-737 (0.1–30 µM) alone (open circle) or together with 2.0 µM Maritoclax (closed circle) and then incubated for 24 h. Cell viabilities were determined by MTT assay. ***B***, UACC903 cells were treated with either 5.0 µM of ABT-737 alone or together with 2.5 µM Maritoclax and then incubated for 24h. Cells were stained with a Live/Dead assay reagent for 30 min and then analyzed through flow cytometer. ***C,*** Effect of Maritoclax on apoptosis. UACC903 cells were treated with either 5.0 µM of ABT-737 alone or in combination with 2.5 µM Maritoclax and incubated for 24h. Cells were stained with Annexin-V-FITC and then analyzed through flow cytometer. ***D-E,*** Melanoma cells UACC903 were treated with 5.0 µM of ABT-737 and 2.5 µM of Maritoclax together for mentioned time points. After incubation, cells were harvested; total lysates were prepared, and fractionated. The Western blot analysis was performed using mentioned antibodies and same blots were re-probed with either other antibodies or with anti-GAPDH to access loading.

Although ABT-737 is the most potent and selective small-molecule Bcl-2 inhibitor, it is not very effective against cancer cells that harbor high levels of Mcl-1, such as breast, lung, and melanoma cells [24]. To determine whether Maritoclax sensitizes melanoma cells to ABT-737, we treated UACC903 cells with increasing doses of ABT-737 alone or in combination with 0.5, 1.0, and 2.0 µM Maritoclax for 24, and 48h (Fig. S1). As a single agent, ABT-737 was not efficient in killing UACC903 cells (IC_50_ >30 µM; Fig. S1 and Fig. 4A; open circle). Addition of small amount of Maritoclax (0.5 and 1.0 µM) enhances the sensitivity of ABT-737 to melanoma cells (Fig. S1 and Fig. 4A; IC_50_ 1.3 µM; close circle). Similarly, Maritoclax potentiated apoptotic effect of ABT-737 from 20% –71% and 18%–62% when measured by Live and Dead and annexin-V assay, respectively (Fig. 4B & C). Furthermore, treatment of ABT-737 resulted in the activation of Mcl-1, followed by suppression of Bcl-2, Bcl-xL (Fig. 4D, left panel). However, in contrast its combination with Maritoclax inhibited Mcl-1 as early as 12h (Fig. 4D, right panel). Earlier studies suggest proteolytic cleavage of Bax with various chemotherapeutic agents [41], [42]. Calpain mediated Bax cleavage to smaller product (18-kDa, Bax/p18) has been shown to induce apoptosis [41]–[43]. We investigated whether treatment of melanoma cells with ABT-737 or in combination induces Bax cleavage. The Bax antibody raised against N-terminal (N-20) detected the full-length Bax protein in both treated and untreated cells (Fig. 4E). Bax (N-20) antibody detected only one band of approximately 21-kDa (Bax/p21). Treatment of ABT-737 decreased the expression of Bax in 24h (Fig. 4E, left panel). Early decrease in Bax expression was observed when cells were treated in combination of ABT-737 and Maritoclax (3h, Fig. 4E, right panel). Another specific monoclonal antibody raised against Leu45 Bax, detected full-length Bax protein (Bax/p21). This antibody also detected a band of 18-kDa (Bax/p18) in treated cells only (Fig.4E, left and right panel). Therefore, similar to the previous reports, we found that Bax/p21 was cleaved, resulting in the production of a p18 fragment [41]-[43]. Activation of caspase-3 and PARP cleavage was observed after Bax cleavage (Fig. 4E, left panel). In contrast combination of Maritoclax resulted in rapid activation of caspase-3 followed by PARP inhibition (Fig.4E, right panel)).

### Maritoclax inhibits growth of 3D melanoma spheroids and sensitizes to ABT-737

**Figure 5:**
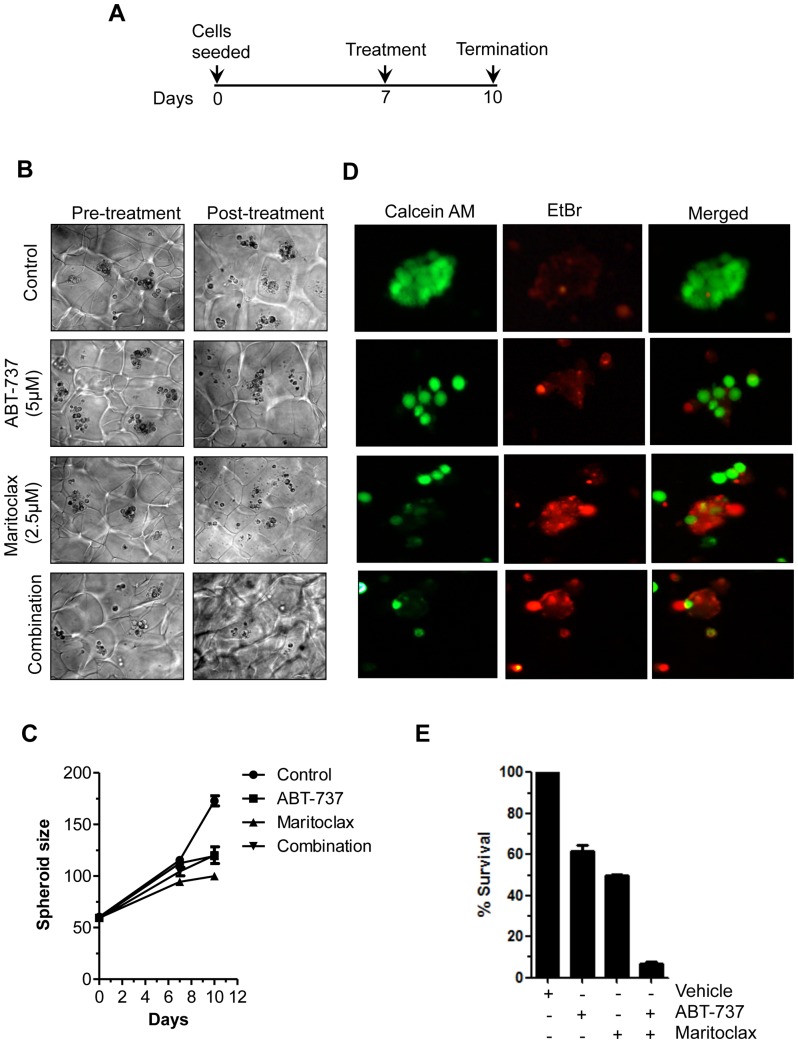
Maritoclax inhibits 3D spheroids and colony forming ability of melanoma cells. *A*, UACC903 cells were grown as spheroids in Algimatrix for seven days and treated with either ABT-737 (5.0 µM) or Maritoclax (2.0 µM) alone or in combination for 3 days and on 10^th^ day experiment was terminated. ***B-C***, After termination of experiments spheroids were counted, size of spheroids was measured by using Image J software as described under Materials and Methods. Graph show corresponding quantification of spheroid growth using image analysis. ***D,*** Spheroids were isolated from matrix using dissolving buffer. Note the decrease of viable cells (calcein-AM) and increase of dead cells (ethidium bromide) in the treated spheroids only. ***E,*** UACC 903 cells were exposed to either 5.0 µM of ABT-737 or 2.5 µM Maritoclax alone or combination for 24h. Cells were re-plated after treatment for clonogenic assay. The effect of treatment was assessed on the basis of percent cell survival in comparison with the controls.

The 3D spheroid model mimics tumor architecture and microenvironment and is used for investigating drug effects on growth, invasion, and viability of melanoma cells, which mimic those of treatment *in vivo*. Melanoma UACC903 cells were allowed to develop spheroids for 7 days in Algimatrix. On day 7 cells were treated with either Maritoclax or ABT-737 or in combination for three days (Fig. 5A). After incubation, size of spheroids was measured and live-dead assay was performed in isolated spheroids. As shown in Fig. 5B and C, Maritoclax inhibited the size of spheroids, as compared to vehicle treated control and ABT-737. When the viabilities of spheroids were considered by live-dead assay, Maritoclax sensitizes these spheroids to ABT-737 as reflected in decreased viable cell staining (calcein-AM) and increased dead cell staining (ethidium bromide) compared with control spheroids (Fig. 5C).

### Maritoclax inhibits colony-forming ability of melanoma cells

The long-term effects of combination treatment with Maritoclax and ABT-737 were assessed by clonogenic assay. As shown in Fig. 5E, treatment of either ABT-737 or Maritoclax alone suppressed colony forming ability of melanoma cells by approximately 40%, however colony formation was reduced to 90% when cells were treated together (Fig. 5E).

Taken together, these data suggest that the pro-apoptotic synergy between Maritoclax and ABT-737 is, at least in part, due to the degradation of Mcl-1 induced by Maritoclax and simultaneous inhibition of Mcl-1 and Bcl-2 may be an effective strategy for melanoma therapy.

## Discussion and Conclusions

Malignant melanoma is highly resistant to chemotherapeutic treatments largely due to an intrinsic resistance of the neoplastic melanocytes to undergo apoptosis. Apoptosis is a highly conserved cellular process activated in response to a variety of stimuli, which culminates in the activation of nucleases and cysteine aspartic acid specific proteases (caspases) responsible of degradation of cellular proteins that ultimately lead to a decay of affected cells. Given the widespread expression of the anti-apoptotic Bcl-2 family member Mcl-1 in human melanoma [11], we hypothesized that Mcl-1 may be one of the key factors influencing the chemo sensitivity of this malignancy. To date, numerous strategies, including small-molecule BH3 mimetics, stapled BH3 peptides, and down-regulation of Mcl-1 by kinase inhibitors, deubiquitinase inhibitors, and antisense oligonucleotides, have been attempted to target Mcl-1 for cancer treatment [44], however, none of the Mcl-1 specific inhibitors are in clinic. Furthermore, none of the small molecule BH3 mimetics reported so far, including ABT-737, obatoclax, BH3-M6, gossypol, and its derivatives, is specific for Mcl-1. Recently, based on competitive stapled peptide screening [45] and structural based design [46] Mcl-1 specific inhibitors have been reported. In the present study we used a specific inhibitor of Mcl-1, Maritoclax [19], to restore the apoptosis signaling pathway in human melanoma. Our results suggest that Maritoclax, along with ABT-737 hold great promise as a new class of therapeutic agents for metastatic melanoma.

Our studies using Maritoclax, demonstrated a strong and specific down-regulation of the Mcl-1 target protein in human melanoma cells. The observation that expression of Bcl-xL, other anti-apoptotic members of the Bcl-2 family [47], was not altered by Maritoclax treatment further reflects the specificity for Mcl-1. We also found that Mcl-1 expression in melanoma cells was differential and highest expression was observed in UACC 903 cells. Interestingly, UACC 903 cells were found more sensitive to Maritoclax. Whether expression of Mcl-1 or interaction between Bcl-2 family members determines the sensitivity of cells to Maritoclax in melanoma is worth to investigate.

We found that Maritoclax antagonizes Mcl-1 by targeting Mcl-1 for proteasome-mediated degradation. Furthermore, Noxa has been shown to negatively regulate Mcl-1 [48], probably by increasing the binding of the E3 ligase Mule and decreasing the binding of the deubiquitinase USP9X to Mcl-1 [49]. Our results suggest that Maritoclax did not significantly affect Noxa protein levels, in accord to previous report [19]. The earlier investigations have shown the significance of Bim and Bmf in homeostasis and disease progression [50] and suggested that both proteins associate with components of the cytoskeleton, that is, microtubules for Bim and actin filaments for Bmf. On certain cellular stresses, such as those caused by UV radiation or loss of adhesion, both proteins were found to change their subcellular localization pattern [51]. Along these lines the role of Mcl-1 degradation and Bim induction has been shown to be associated with anoikis [52]. Bim is tightly regulated by transcriptional and post-transcriptional events [53]. Activation of JNK signaling [54], cAMP/PKA pathways [55] has been implicated with the expression level of Bim. Along these lines, recently it was shown that ABT-737 induces Bim expression by JNK signaling pathways [56]. We found that degradation of Mcl-1 is associated with the higher expression of Bmf and Bim, release of these proteins from cytoskeleton to mitochondria. How Maritoclax induces the expression of Bim and Bmf remains to be elucidated. Since Maritoclax has been shown to bind actin [57], we further investigated whether induced release of Bim and Bmf is associated with Mcl-1 degradation. Interestingly, actin polymerization inhibitor cytochalasin D, had no effect on Mcl-1 degradation, caspase activation and PARP cleavage suggesting that Maritoclax- induced release of Bmf and Bim is likely to be secondary effect (cell detachment) and is unlikely to be due to actin binding (cytoskeleton damage). This also explains the observed higher expression of Bax and Bak in Maritoclax treated cells and also provides the importance of Mcl-1 degradation in the apoptotic response. Together, these results suggest a model for apoptosis where Mcl-1 degradation is required as a priming event to sensitize the cell towards Bax induction, which occurs in a manner that is dependent on the BH3-only protein Bim induction.

The poor sensitivity of several tumors to BH3-mimetic ABT-737 has been linked with the expression of Mcl-1 suggesting that elevated expression of Mcl-1 will suffice for resistance to ABT-737 [24], [30]. Similarly, in this study, we observed that ABT-737 as a single agent resulted in an increase in Mcl-1 protein levels, which was associated with the acquired resistance to ABT-737; however, co-treatment with a suboptimal dose of Maritoclax (2.5 µM) abolished Mcl-1 accumulation and markedly enhanced ABT-737 sensitivity. Our results showed that treatment of cells with either ABT-737 alone or in combination with Maritoclax preceded to Bax cleavage. This is in accord with the previous findings, regarding the cleavage of Bax [41]–[43]. Previously, a late or early Bax cleavage was shown to be mediated through Calpain [41]–[43], whether ABT-737 and Maritoclax induced Bax cleavage is mediated through same mechanism entails further investigation.

Using 3D spheroid model, we showed that combination of Maritoclax with ABT-737 is an effective strategy to treat melanoma. Treatment of Maritoclax alone reduces the size of spheroids as compared to ABT-737. Unexpectedly, size of spheroids was larger when treated in combination of ABT-737 and Maritoclax. Whether ABT-737 modulates Maritoclax effects’ is not clear, nonetheless when viabilities of spheroids were analyzed by live-dead assay, Maritoclax sensitizes spheroids to ABT-737. Taken together, these results support the notion that combination or sequential treatment with Mcl-1 inhibitors may be necessary for ABT-737 to achieve maximum therapeutic efficacy and avoid or overcome drug resistance. Similarly, melphalan and flavopiridol, both are reported to repress Mcl-1 in lung carcinoma and myeloma [58], [59]. Furthermore, Mcl-1 inhibition not only potentiates apoptotic effects of BH3 mimetic ABT-737, but may also be useful to synergize other therapeutic tools [26], [60]. Thus, Mcl-1 may underlie resistance to several forms of pro-apoptotic signals, and hence is a valuable therapeutic target for treatment of melanoma [61].

In conclusion, our results indicate that addition of Maritoclax to BH3-mimetics or to current drug regimens with BH3-mimetics are avenues worth exploring for melanoma therapy, particularly if dosing schedules can be developed to maintain the most favorable balance between Mcl-1 and NOXA activity over time. Thus, the combination therapy represents a promising new treatment strategy for malignant melanoma that calls for further investigation in preclinical models.

## Supporting Information

Figure S1 **UACC903 cells were treated with increasing amount of ABT-737 (0.1-30 µM) alone or together with 0.5, 1.0, and 2.0 µM Maritoclax and then incubated for 24 and 48h.** Cell viabilities were determined by MTT assay.
(TIF)
