# Supplementary figures and images for: Proteasomal Degradation of Mcl-1 by Maritoclax Induces Apoptosis and Enhances the Efficacy of ABT-737 in Melanoma Cells
Source: PLoS One. 2013 Nov 4;8(11):e78570. doi: 10.1371/journal.pone.0078570 (PMC3817219; doi:10.1371/journal.pone.0078570)

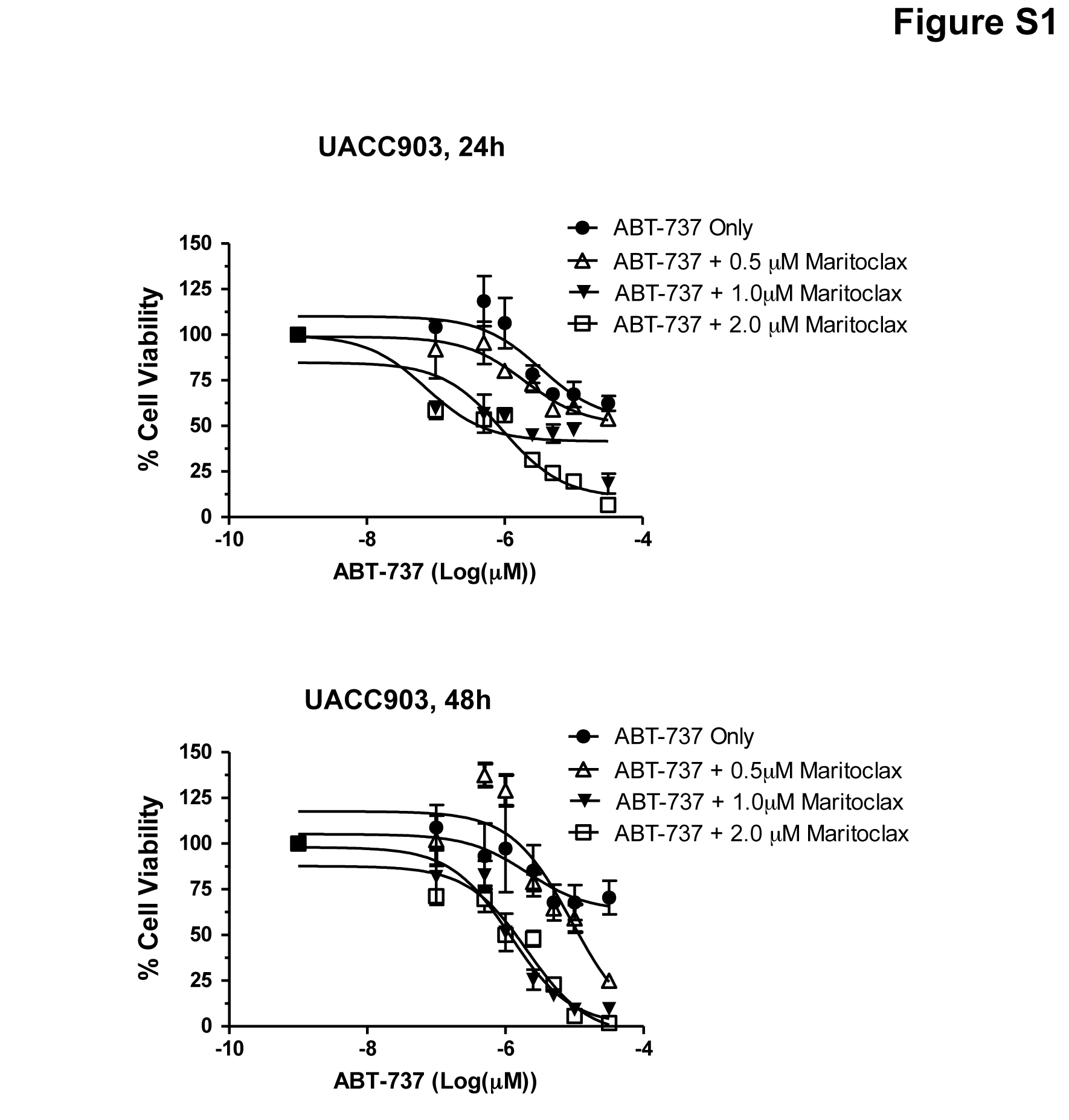

Supplement: Figure S1 — UACC903 cells were treated with increasing amount of ABT-737 (0.1-30 µM) alone or together with 0.5, 1.0, and 2.0 µM Maritoclax and then incubated for 24 and 48h. Cell viabilities were determined by MTT assay. (TIF) [file pone.0078570.s001.tif]
